# Primary mucosal melanoma of the sinonasal tract: Report of 18 patients and analysis of 1077 patients in the literature

**DOI:** 10.1590/S1808-86942011000100010

**Published:** 2015-10-19

**Authors:** Bijan Khademi, Hajar Bahranifard, Hamid Nasrollahi, Mohammad Mohammadianpanah

**Affiliations:** 1 Dr. (Professor of Otolaryngology, Department of Otolaryngology, and Head and neck surgery, Khalili Hospital, Shiraz University of Medical Sciences, Shiraz 71936-13311, Iran); 2 Dr. (Resident of Otolaryngology, Department of Otolaryngology, and Head and neck surgery, Khalili Hospital, Shiraz University of Medical Sciences, Shiraz 71936-13311, Iran); 3 Dr. (Resident of Radiation Oncology, Department of Radiation Oncology, Nemazee Hospital, Shiraz University of Medical Sciences, Shiraz 71936-13311, Iran); 4 Dr. (Head of Radiation Oncology Department,, Nemazee Hospital, Shiraz University of Medical Sciences, Shiraz 71936-13311, Iran)

**Keywords:** surgery, chemotherapy adjuvant, melanoma, mucous membrane, head and neck neoplasm, radiotherapy

## Abstract

The present study aimed at reporting on the characteristics, prognostic factors and treatment outcomes of 18 cases of nasosinusal mucosa melanoma, and do a literature review on the subject.

**Methods:** between 1995 and 2005, 18 patients consecutively diagnosed with nasosinusal mucosa melanoma were managed in our institution. We reviewed the literature in PubMed and Scopus in order to find the main series from studies associated with this topic. We found a total of 35 series, involving 1,077 patients with nasosinusal mucosa melanoma.

**Results:** we found 16 men and 2 women, with age at presentation between 51 and 80 years (median of 58 years). All these patients were submitted to surgical excision followed by radiotherapy with or without chemotherapy. The survival median was 15 months, and the 5-year general survival had a percentage value of 23%. Considering our univariate analysis: tumor staging and complete remission after initial treatment were deemed relevant prognostic factors. Nonetheless, considering the multivariate analysis, only disease stage was statistically significant.

**Conclusion**: nasosinusal melanoma is a rare and aggressive tumor, with high loco-regional and distant failure rates, and poor treatment outcomes. Notwithstanding, in a literature review we found significant improvements considering the 5-year survival for recent series when compared to previously reported ones.

## INTRODUCTION

Mucosal melanoma is a highly aggressive tumor arising from the mucosa of the head and neck, anorectal and genitourinary system. The incidence of mucosal melanoma is believed to be stable, contrary to its counterpart the cutaneous melanoma that has been rapidly increasing. Primary mucosal melanoma of the head and neck is a rare entity, occurring much less frequently that its cutaneous relatives and accounts for 1.3% of all malignant melanomas. However; head and neck region is the most frequent primary site for mucosal melanomas and consists of 55.4% of all mucosal melanomas.^1^ Among head and neck anatomical locations, sinonasal tract is a common primary site for this malignancy.^2,^ ^3^ The mean age of presentation in patients with sinonasal mucosal melanoma is approximately 67 years, but it can occur at any age group and is extremely rare in ages below 30.^1^ It has been suggested to have a slight predilection for males as reported by some authors.^4^ Approximately 19-26.6% of patients with head and neck mucosal melanomas have positive regional lymph nodes at presentation.^1,^ ^4^ The presence of positive lymph nodes in sinonasal mucosal melanomas has been considered as a poor prognostic factor in some studies.^1,^ ^5,^ ^6^ The treatment of choice in mucosal malignant melanoma is surgery followed by radiotherapy, particularly in cases of small or doubtful margins of resection.^7^ The prognosis of mucosal malignant melanoma is poor with 0% to 50% of 5-year survival.^1,^ ^7,^ ^8^ Herein, we describe 18 cases of sinonasal mucosal melanoma and review the major previous reported series including more than 1000 patients with sinonasal mucosal melanoma.

## METHODS

The study was based on the data collection of Radiation Oncology Department, Namazi Academic Hospital, Shiraz University of Medical Sciences.This retrospective study was performed by reviewing patients' records from our departmental computer databases or from written patient lists. Eighteen consecutive patients diagnosed with primary mucosal melanoma of the sinonasal tract who were treated and followed up in our institution between 1995 and 2005, were selected for the present study. The study included all patients with histopathologically proven diagnosis of mucosal melanoma of the sinonasal tract. The diagnoses of the cases were confirmed by the presence of melanin and/or immunoreactivity to a melano associated markers (S-100 and HMB-45). Metastatic lesions arising from disseminated cutaneous or mucosal melanomas of any other site were excluded. The staging of all cases was determined according to the following basic staging system: The lesions confined to the primary site were defined as stage I, regional cervical lymph node involvement as stage II, and distant metastasis as stage III. Clinical data were reviewed to gather the patients' characteristics, prognostic factors and treatment outcomes. We performed a literature review of PubMed and Scopus using the search terms of “mucosal melanoma” and “sinonasal” and/or “nasal cavity” and/or “paranasal sinuses” to find out the references for the present study. Case series including at least 6 cases over the last 20 years were included for the present review. All case reports and few articles in non-English language or with unavailable full text were excluded. In all, we found 35 series including 1077 patients with sinonasal mucosal melanoma.

## RESULT

The medical records of 18 patients diagnosed as having mucosal melanoma of the sinonasal tract, and treated at our institution between 1995 and 2005, were reviewed. This number contributed 12% of all sinonasal malignancies and 2.6% of all malignant melanomas in the study period. The mean and median age at diagnosis was 65 years (range 51- 80 years), and 16 (87/5%) were men. The most common symptoms at presentation were epistaxis and nasal obstruction and discharge. Nasal cavity (62%) was the most frequent primary site, followed by ethmoidal sinuses (19%) and maxillary sinuses (19%). The median tumor size was 4 cm (range 2-7 cm). Based on the common basic staging system for the mucosal melanoma, eight patients had stage I (no evidence of regional or distant metastasis) 7 patients had stage II (regional lymph node metastasis) and 3 patients had stage III (distant metastatic disease). A combination of excision followed by external beam radiotherapy was used to treat all cases. All patients received external beam radiotherapy using cobalt-60 units or 9 MV X-ray photons from a linear accelerator. A mean dose of 53 Gy (range 20-70 Gy) was delivered via a daily fraction of 2 Gy, with five fractions per week. Five (28%) patients received a median 2 (range 1-6) cycles of single-agent chemotherapy with dacarbazine 850 mg/m^2^.

At the last follow-up, 6 cases (33%) were alive with no evidence of disease (follow-up range, 3-65 months), 9 cases (50%) died of disease and the median time to death for these patients were 15.6 months (range, 15-17 months) and 3 patients (17%) are alive with disease. The overall cancer specific survival rate was 68.7% at 1 year, 45.8% at 2 years and 22.9% at 5 years. Disease-free survival was 51.3% at 1 year, 25.6% at 2 years and 12.8% at 5 years. The median survival was 15 months (range 3-65 months). Local recurrence was observed in 9 (50%) patients; 3 (16.7%) cases had a recurrence in the first 6 months after treatment, and 9 (50%) cases had a recurrence 6 months to 3 years after treatment. The median time to local recurrence was 11 months (range 2-14 months). Distant metastases developed in 3 patients (16.7%). The median time from diagnosis of sinonasal melanoma to distant metastases was 6 months (range 7-9 months) and the median time to death after metastasis was 6 months. Various prognostic factors including age, sex, stage of disease, initial complete remission, total dose of radiotherapy and the size of primary tumor were analyzed to establish their effect on patient overall survival. On univariate analysis for overall survival, stage of disease (hazard ratio for death = 5.794; 95 per cent CI = 1.645-20.408; *p* = 0.006) and initial complete remission to the treatment (hazard ratio = 13.409; 95 per cent CI = 1.565-114.920; *p* = 0.018) were prognostic factors. On multivariate analysis, only stage of disease (hazard ratio = 22.170; 95% CI = 1.660-296.061; p = 0.019) retained statistical significance.

## DISCUSSION

Malignant neoplasms of the sinonasal tract are rare and account for about 3% of head and neck malignant tumors. Sinonasal malignancies comprise a heterogeneous and biologically diverse group of neoplasms occurring in the sinonasal tract.^9,^^10,^^11^ Primary sinonasal mucosal melanoma is a rare entity, which constitutes about 1.5-9% of all malignancies in this site.^12,^ ^13^ Sinonasal melanoma tends to have a high rate of locoregional and distant failure and to carry poor outcome with a median survival of 9-52 months and an overall 5-year survival rate of 0% to 50%.^4^, ^5^, ^6^, ^7^, ^8^ ^14^, ^15^, ^16^, ^17^, ^18^, ^19^, ^20^, ^21^, ^22^, ^23^, ^24^, ^25^, ^26^, ^27^, ^28^, ^29^, ^30^, ^31^, ^32^, ^33^, ^34^, ^35^, ^36^, ^37^, ^38^, ^39^, ^40^, ^41^, ^42^ To date, more than one thousand cases of sinonasal melanomas have been reported in the literature.^4^, ^5^, ^6^, ^7^, ^8^ ^14^, ^15^, ^16^, ^17^, ^18^, ^19^, ^20^, ^21^, ^22^, ^23^, ^24^, ^25^, ^26^, ^27^, ^28^, ^29^, ^30^, ^31^, ^32^, ^33^, ^34^, ^35^, ^36^, ^37^, ^38^, ^39^, ^40^, ^41^, ^42^ Sinonasal mucosal melanoma is a tumor that usually occurs in the seventh decade of life and rarely before the fourth decade, as found in our study. In the present study, the mean and median age of our patients was 65 years, which is consistent with the results of the literature review in which average mean age of 683 patients in the reported series was 64.5 years. [Table 1] There are conflicting reports regarding the incidence of sinonasal mucosal melanoma in males and females.^6,^ ^8,^ ^26,^ 40, 41, 42, 43 The male/female ratio of our patients was 8, which is consistent with the study by Richtig et al.^6^ However, in the literature review, by analyzing the data of the reported series, the mean male/female ratio of 843 patients was 1.07 and we did not find significant differences. [Table 1]

**Table 1:** Patients' characteristics and median and 5-year overall survival in major reported series

Authors	No of patients	Male/Female ratio	Mean age	Median survival (month)	5-year overall survival (%)
Manolidis^4^	13	1,1	71	31	14
McLean^5^	22	-	67	12	10
Richtig^6^	9	8	62	30	-
Ziółkowska ^8^	6	0,2	69	-	50
Huang^14^	15	1,1	69	23	33
Wagner^15^	17	2,1	68	-	28
Dauer^16^	61	1	67	-	22
Pomar Blanco^17^	6	0,5	70	-	33
Peng^18^	44	-	-	24	25
Martin^19^	20	0,7	77	23	5
Prasad^20^	59	1	63	36	35
Temam^21^	46	1,1	58	-	20
Thompson^22^	115	0,9	64	28	35
Oueslati^23^	10	1	58	18	6
Slavícek^24^	13	-	-	9	-
Ryuto^25^	12	0,5	61	20	22
Diaz Molina^26^	17	0,4	74	-	36
Cheng^27^	23	2,3	68	20	22
Regauer^28^	14	-	68	9	-
Folz^29^	28	-	67	-	46
Brandwein^30^	25	0,7	65	39	40
Bridger^31^	27	1,3	66	52	46
Patel^32^	35	1,3	63	-	47
Faye-Lund^33^	13	0,6	72	-	-
Nandapalan^34^	178	-	-	-	45
Kingdom^35^	17	-	-	-	20
Lund^36^	58	1,1	64	21	28
Bachar^37^	49	0,5	68	28	29
Gaze^38^	23	0,6	69	14	-
Guzzo^39^	26	2,4	58	20	21
Owens^40^	11	4,5	55	-	33
Hyodo^41^	14	0,4	64	-	20
Marco Meleti^42^	25	0,7	67	-	0
Kumar^43^	10	2,3	51	25	40
Nakaya^44^	16	2,2	65	-	31
Present study	18	8	65	15	23
Total	1095	1,07	64,5	25,8	31

Epistaxis and nasal obstruction were the most frequent presenting symptoms in our and most other reported series.^4,^ ^14,^ ^16,^ ^27,^ ^36,^ ^43^ There are divergent reports concerning the rate of cervical lymph node involvement at presentation in patients with sinonasal melanomas. The incidence of positive lymph nodes in our study was 44% (all patients with stage II and one patient with stage III). In a review by Manolidis, 18.7% of patients with malignant mucosal melanoma of the head and neck presented with cervical lymph node metastasis.^4^ In the national cancer data base report on cutaneous and noncutaneous melanoma by Chang et al, this incidence was 26.6%.^1^ In the reported series by Temam et al, only 5 (11%) patients had positive lymph node at presentation.^20^ In contrast, Guzzo reported 52% of patients with regional lymphatic metastasis at the time of diagnosis.^38^ Furthermore, there are conflicting reports on the subject of the prognostic impact of regional lymph node metastasis at presentation on survival. Shah et al concluded that regional lymphatic metastases do not impact on the survival in patients with mucosal melanoma of the head and neck.^43^ However; most large reported series demonstrated that the presence of positive lymph nodes in these patients have a negative influence on disease free and overall survival.^1,^ ^21,^ ^27,^ ^37^ In a review by Manolidis, the 5-year overall survival of patients with positive lymph node was 21.4% against 30% for patients with negative lymph node.^4^

The majority of patients with mucosal melanoma present in stage I disease. Based on the review of the reported series by Manolidis, of 547 patients, 75.3% presented with stage I, 18.1% with stage II and 6.6 % with stage III. In the present study, these values were 44% for stage I, 39% for stage II and 17% for stage III. Sinonasal mucosal melanoma tends to have high rate of locoregional recurrence following the initial treatment. Despite large proportion of patients presented in stage I disease, the rates of locoregional recurrence and distant metastases are high. As mentioned by Manolidis, of 484 patients in 14 series, 53.3% developed locoregional recurrent disease. In addition, Manolidis found an average of 51.5% distant failure among 332 patients with mucosal melanoma.^4^ In agreement with the findings of this review, most recent studies found a rate of 40% to 85% for locoregional recurrent disease. Most locoregional failure occurred within the first 24 months after the treatment.^2,^ ^6,^ ^7,^ ^15,^ ^17,^ ^25,^ ^32,^ ^40,^ ^42^ Furthermore, 25% to 77% of all patients in recent reported series developed distant failure following the initial treatment. Similarly, the most distant failures occurred within 2 years.2, 6, 7, 14, 15, 17, 25, 32, 40, 42

The median time from locoregional recurrent disease to death is 19-75 months in reported series. In addition, the median time from distant failure to death is 3-20 months in these studies.^2,^ ^7,^ ^16,^ ^18,^ ^19^ In our study, the median times to local and distant failure were 11 months and 6 months, respectively. Moreover, the median times from local and distant failure to death were 12 and 6 months, respectively. According to the pooled data from 20 series, we found a median 25.8 months for 605 patients with sinonasal mucosal melanoma. In addition, by analyzing the pooled data from 31 series, we found an overall 5-year survival rate of 31% for 1023 patients in these series. Although this 5-year survival is poor, it shows a significant improvement compared with the results of the previous literature review by Manolidis, in which the 5-year overall survival was 17.1%. Part of this survival improvement may be due to use of multimodality therapy for these patients.^4^ The median survival of our patients was 15 months and with a 5-year overall rate of 23%. The lower median and 5-year overall survival rates of our patients may be due to a larger proportion of cases with stage II and III compared with other series. Optimal treatment of sinonasal mucosal melanoma remains a challenge. Complete surgical excision is the mainstay of definitive therapy for localized disease with or without regional lymph node metastases.^3^ Postoperative radiotherapy is usually considered for the majority of patients with sinonasal mucosal melanoma.^16,^ ^34,^ ^40^ The role of postoperative adjuvant radiotherapy in the treatment of sinonasal mucosal melanoma remains unclear.^44^, ^45^, ^46^ Several reports concluded that the addition of radiotherapy to surgical excision conferred a local control advantage even for patients with small tumors. Additionally, there are a few reports, which suggest an improvement in survival for patients receiving postoperative radiotherapy.^35,^ ^47^ Although most reported series and reviews found no survival benefit from postoperative radiotherapy,^2,^ ^19,^ ^21,^ ^34,^ ^40^ many authors recommended aggressive local therapy with adjuvant or salvage radiotherapy for patients with sinonasal mucosal melanoma, even in the absence of a survival benefit.^36,^ ^48,^ ^49^ Despite aggressive locoregional therapy, the majority of patients with sinonasal mucosal melanoma eventually die from distant failure and the outcomes for these patients in almost all studies have remained dismal. Management of disseminated melanoma is a challenge because of a paucity of effective systemic treatment.^50^ In an effort to improve systemic disease control and survival, adjuvant and palliative chemotherapy has been widely used.^3^ Dacarbazine monochemotherapy with a single dose of 850-1000 mg/m^2^ or a multiple dose regimen of 250 mg/m^2^ for 5 days per cycle is currently considered the standard first-line treatment in patients with advanced malignant melanoma.^51,^ ^52^ The conventional systemic treatment modalities such as chemotherapy and immunotherapy are disappointing.^53^ Current studies have been focused on the molecular-based therapeutic strategies to find out the novel and emerging compounds such as growth factor and enzyme inhibitors, and antiangiogenic and immunomodulatory drugs.^54^

Prognostic variables were evaluated in many reported series. On univariate analysis, the size and location of primary tumor, stage of disease, sex, melanosis, postoperative radiotherapy and complete remission have been prognostic factors in some studies. However, on multivariate analysis, melanosis, younger age (less than 50 years) and early disease stage were favorable prognostic factors for overall survival.^16,^ ^21,^ ^37,^ ^46^ In the present study, on univariate analysis for overall survival, stage of disease and initial complete remission to the treatment were prognostic factors. On multivariate analysis, only stage of disease retained statistical significance.

## CONCLUSION

The patients' characteristics, prognostic factors and outcome of this study were consistent with the most recent reported series. Based on the findings of the present study and those of previous research in the literature, we found that sinonasal mucosal melanoma is a rare, but highly malignant tumor. This aggressive tumor mostly affected patients in the 6^th^ to 8^th^ decades of life with no sex predilection. Sinonasal mucosal melanoma tends to have a high rate of locoregional and distant failure and to carry poor outcome after the current treatment and with a median survival of 25.8 months and overall 5-year survival rates of 31%. However, this review shows significant improvement in terms of 5-year survival for recent series compared with previously reported series. Complete surgical excision is the mainstay of definitive therapy for locoregional disease. Postoperative adjuvant radiotherapy improves the locoregional control in patients with sinonasal mucosal melanoma. The impact of adjuvant radiotherapy and chemotherapy on survival remains to be defined.
